# Cortical Tracking of Complex Sound Envelopes: Modeling the Changes in Response with Intensity

**DOI:** 10.1523/ENEURO.0082-19.2019

**Published:** 2019-06-27

**Authors:** Denis P. Drennan, Edmund C. Lalor

**Affiliations:** 1 School of Engineering, Trinity Centre for Biomedical Engineering and Trinity College Institute of Neuroscience, Trinity College Dublin, Dublin 2, Ireland; 2 Department of Biomedical Engineering, Department of Neuroscience, and Del Monte Institute for Neuroscience, University of Rochester, Rochester, NY 14627

**Keywords:** auditory evoked potential, deconvolution, electroencephalography, encoding model, envelope tracking, speech

## Abstract

Characterizing how the brain responds to stimuli has been a goal of sensory neuroscience for decades. One key approach has been to fit linear models to describe the relationship between sensory inputs and neural responses. This has included models aimed at predicting spike trains, local field potentials, BOLD responses, and EEG/MEG. In the case of EEG/MEG, one explicit use of this linear modeling approach has been the fitting of so-called temporal response functions (TRFs). TRFs have been used to study how auditory cortex tracks the amplitude envelope of acoustic stimuli, including continuous speech. However, such linear models typically assume that variations in the amplitude of the stimulus feature (i.e., the envelope) produce variations in the magnitude but not the latency or morphology of the resulting neural response. Here, we show that by amplitude binning the stimulus envelope, and then using it to fit a multivariate TRF, we can better account for these amplitude-dependent changes, and that this leads to a significant improvement in model performance for both amplitude-modulated noise and continuous speech in humans. We also show that this performance can be further improved through the inclusion of an additional envelope representation that emphasizes onsets and positive changes in the stimulus, consistent with the idea that while some neurons track the entire envelope, others respond preferentially to onsets in the stimulus. We contend that these results have practical implications for researchers interested in modeling brain responses to amplitude modulated sounds.

## Significance Statement

A key approach in sensory neuroscience has been to fit linear models to describe the relationship between stimulus features and neural responses. However, these linear models often assume that the response to a stimulus feature will be consistent across its time course, but just scaled linearly as a function of the stimulus feature’s intensity. Here, using EEG in humans, we show that allowing a linear model to vary as a function of the stimulus feature’s intensity leads to improved prediction of unseen neural data. We do so using both amplitude modulated noise stimuli as well as continuous natural speech. This approach provides more robust measures of envelope tracking and facilitates the study of its underlying mechanisms.

## Introduction

Characterizing how the brain responds to stimuli has been a major goal of sensory neuroscience for decades (Hubel and Wiesel, 1962). One key approach to this problem has been to fit models to describe the relationship between sensory inputs and neural responses (Wu et al., 2006). Our understanding of the sensory system can then be assessed by quantifying how well such models predict neural responses to novel stimuli (Carandini et al., 2005).

A central feature of such models has been a “linear receptive field” stage that seeks to account for some of the neural response as a linear weighted sum (i.e., a linear filter) of particular features of the sensory input (e.g., the contrast of a visual stimulus across space or the amplitude of an acoustic stimulus across time and frequency). In neural spiking models, this linear filtering stage is typically just one of several stages (e.g., linear, nonlinear, and Poisson) that seek to capture how stimulus variations are reflected in spike trains (Chichilnisky, 2001). However, with macroscopic data like fMRI (Boynton et al., 1996) or EEG/MEG (Crosse et al., 2016), this linear filtering stage often represents the entirety of the model. Implicitly (or explicitly), these linear models assume that responses to a stimulus feature will be temporally and morphologically consistent across its time course, but just scaled linearly as a function of the stimulus feature’s intensity. In other words, they assume that responses to a particular stimulus feature can be modeled by a linear impulse response function.

In auditory neuroscience these filters have been viewed as representing the spectrotemporal receptive fields (STRFs) of auditory cortical neurons (Aertsen and Johannesma, 1981). They are often fit using audio stimuli with broad spectrotemporal statistics so as to characterize how neurons might respond to any sound (Depireux et al., 2001), although there has been increasing interest in the use of more naturalistic stimuli such as animal vocalizations (Theunissen et al., 2001; Machens et al., 2004) and human speech. The latter has included efforts to fit linear response functions between various speech features (e.g., envelope, spectrogram, phonemes, or phonetic features) and population responses in animals (David et al., 2007; Mesgarani et al., 2008), as well as macroscopic measures in humans (Lalor and Foxe, 2010; Ding and Simon, 2012; Di Liberto et al., 2015).

One explicit use of this linear modeling approach has been the fitting of so-called temporal response functions (TRFs) to describe how EEG is affected by variations in visual (Gonçalves et al., 2014) or auditory (Lalor et al., 2009) stimuli. This includes univariate TRFs that model how EEG changes based on a single stimulus feature (e.g., an envelope), and multivariate TRFs that simultaneously model responses to multiple features (e.g., a spectrogram; Ding and Simon, 2012; Di Liberto et al., 2015). In both cases, however, it is typically assumed that changes in the intensity of the stimulus feature produce variations in the magnitude but not the latency or morphology of the responses. While this assumption may be reasonable for certain brain responses in certain brain areas to certain stimulus features (Boynton et al., 1996), there is definitive evidence that it is imperfect for EEG-based TRFs.

One such piece of evidence is the long-known relationship between auditory stimulus amplitude and response latency (Beagley and Knight, 1967). Specifically, while there is a monotonic (although not necessarily linear) relationship between auditory stimulus amplitude and response magnitude, there is also an inverse relationship between stimulus amplitude and response latency. Therefore, modeling neural responses to an ongoing auditory stimulus using a linear univariate TRF is likely to be suboptimal given that it ignores the dependence of response latency (and morphology) on stimulus amplitude.

Here, we aim to demonstrate that by allowing the stimulus-response model to vary as a function of the stimulus amplitude, we can improve the modeling of responses to continuous auditory stimuli. To do so, we propose a simple extension to the standard linear TRF estimation approach that involves amplitude binning a single feature, namely the envelope, and then using it to fit a multivariate TRF. This should allow the TRF to vary across the different amplitude ranges, thus enabling it to account for associated changes in response magnitude, latency, and morphology. We aim to validate that this represents an improved model by comparing how well it predicts EEG data relative to more standard univariate models and discuss other methods that can be used to improve model performance.

## Materials and Methods

EEG data from two experiments were used in this study: one acquired in response to amplitude-modulated broadband noise (AM BBN), the other in response to continuous natural speech (Natural Speech Dataset from https://doi.org/10.5061/dryad.070jc, including amplitude envelopes; Broderick et al., 2018).

### Subjects

A total of 13 subjects participated in the AM BBN experiment; five male, aged 23–35 years; 19 subjects participated in the speech experiment; 13 male, aged 19–38 years, although data from two subjects were later excluded because of uncertainties in response timing due to differences in their data acquisition setup. All subjects had self-reported normal hearing. The protocol for both studies was approved by the Ethics Committee of the Health Sciences Faculty at Trinity College Dublin, Ireland, and all subjects gave written informed consent.

### Stimuli

As mentioned, this study involved experiments using two different types of stimuli, AM BBN and continuous natural speech.

**Figure 1.**
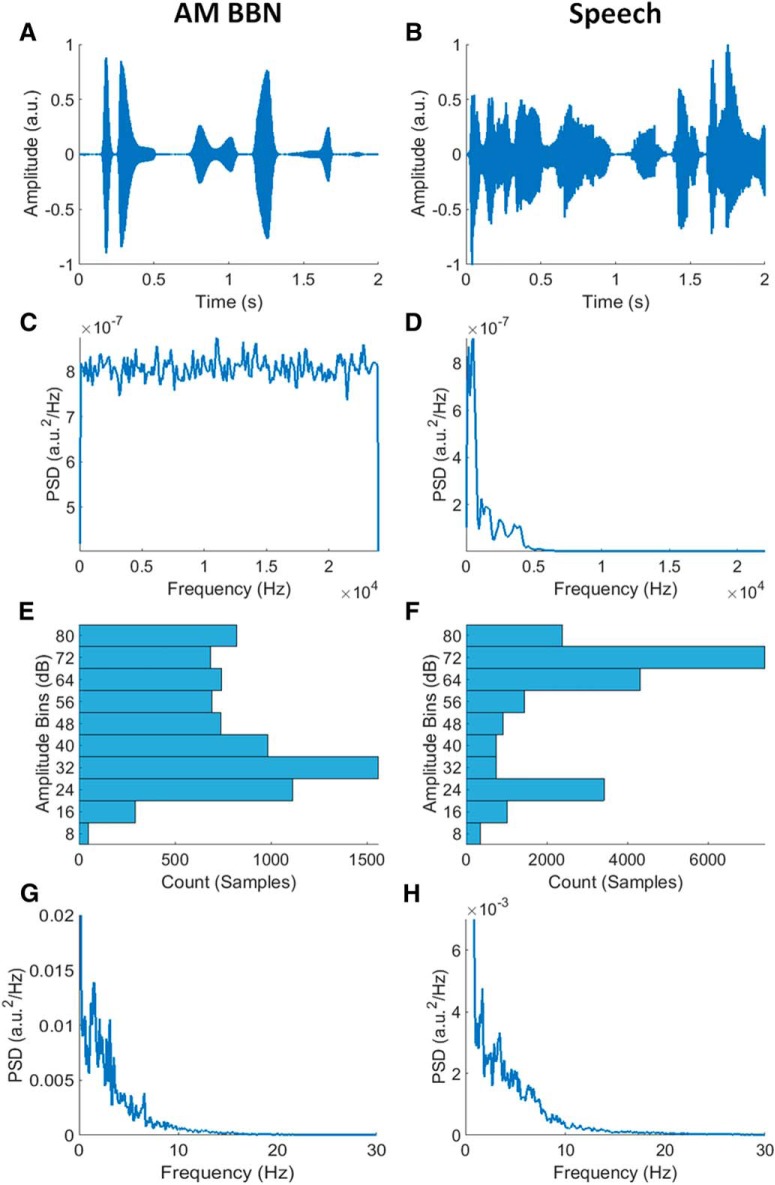
***A***, ***B***, Example segments of AM BBN and speech stimuli, respectively. ***C***, ***D***, Power spectral densities (PSDs) of AM BBN and speech stimuli, respectively. The AM BBN had a broadband frequency distribution by design, while the male speaker had a frequency distribution that was dominated by frequencies below 5000 Hz. ***E***, ***F***, Amplitude histograms of AM BBN and speech envelopes, respectively. Both envelopes had quite broadly distributed amplitude distributions. Please note that the amplitude distribution of the AM BBN envelope was uniform by design, but after extracting the envelope from the AM BBN signal using the Hilbert transform, it was less so. Also note that the amplitude distribution of the speech envelope was more skewed, with a higher percentage of samples in the higher amplitude bins. ***G***, ***H***, PSDs of AM BBN and speech envelopes, respectively. Both signals had envelopes with a bottom-heavy (right-skewed) frequency distribution indicating that their modulation rates were dominated by low frequencies.

The carrier signal for the AM BBN stimulus was uniform broadband noise with energy limited to a bandwidth of 0–24,000 Hz. Its modulating signal (envelope) had a log-uniform amplitude distribution (by design, although less so after envelope extraction, please see below) and a bottom-heavy (right-skewed) frequency (modulation rate) distribution (Fig. 1), so chosen as it has been shown that auditory cortical areas tend to be most sensitive to AM BBN presented at lower modulation frequencies (Liégeois-Chauvel et al., 2004). The envelope was created by first generating a signal with discrete amplitude values with the desired statistical properties, and then interpolating between those discrete points to provide a smooth transition from one modulation amplitude to the next.

The speech stimulus had a bottom-heavy (right-skewed) frequency distribution with energy limited to a bandwidth of 0–22,050 Hz. Its envelope had a log-top-heavy (left-skewed) amplitude distribution, and a bottom-heavy (right-skewed) frequency (modulation rate) distribution, similar to that of the AM BBN stimulus (Fig. 1). It comprised extracts from a professional audio-book version of a popular mid-20th century American work of fiction (i.e., *The Old Man and the Sea* by Ernest Hemingway) written in an economical and understated style and read by a single male American speaker.

### Experimental procedure

In the AM BBN experiment, subjects were presented with 80 repetitions of the same 60-s long AM BBN stimulus as they reclined in a comfortable chair, in a quiet, darkened room, and watched a silent animated cartoon presented on a tablet computer. They were asked *not* to attend to the auditory stimuli, which were presented monaurally to their right ear at a peak level equivalent to that of a 1-kHz pure-tone at 80-dB SPL, using a Sound Blaster X-Fi Surround 5.1 Pro external sound card, a TPA3118D2EVM amplifier, and electromagnetically shielded Etymotic Research ER-2 earphones, via VLC Media Player from VideoLan (http://www.videolan.org). Compensation for the 1-ms sound-tube delay introduced by the ER-2 earphones was applied *post hoc*.

In the speech experiment, subjects were presented with 28 trials of ∼155-s long audiobook extracts. The trials preserved the storyline, with neither repetitions nor discontinuities. Subjects sat in a comfortable chair, in a quiet, darkened room, and were instructed to maintain visual fixation on a crosshair centered on a computer monitor, and to minimize eye blinking and all other motor activities for the duration of each trial. They were asked to attend to the auditory stimuli, which were presented diotically at a comfortable listening level, using Sennheiser HD 650 headphones, via Presentation software from Neurobehavioral Systems. For the purposes of analysis, all trials were truncated to 150 s, and a peak level of 80-dB SPL was estimated (as the original presentation level was not available).

### EEG acquisition

In the AM BBN experiment, 40 channels of EEG data were recorded at 16,384 Hz (analog –3-dB point of 3276.8 Hz), using a BioSemi ActiveTwo system. A total of 32 cephalic electrodes were positioned according to the standard 10–20 system. A further eight non-cephalic electrodes were also collected although only two, those over the left and right mastoids, were used in the analysis. Triggers indicating the start of each 60-s trial were encoded in a separate channel in the stimulus WAV file as three cycles of a 16-kHz tone burst. These triggers were interpreted by custom hardware before being fed into the acquisition laptop for synchronous recording along with the EEG.

In the speech experiment, 130 channels of EEG data were recorded at 512 Hz (analog –3-dB point of 409.6 Hz), using a BioSemi ActiveTwo system. 128 cephalic electrodes were positioned according to the BioSemi Equiradial system, with another two electrodes located over the left and right mastoids. Triggers indicating the start of each ∼155-s trial were presented using Neurobehavioral Systems Presentation software for synchronous recording along with the EEG.

### EEG preprocessing

The EEG data were first resampled to 128 Hz using the *decimate* function in MATLAB (MathWorks). The *decimate* function incorporates an 8^th^ order low-pass Chebyshev Type I infinite impulse response (IIR) anti-aliasing filter. This filter was applied with a cutoff frequency of 64 Hz and was implemented using the *filtfilt* function, ensuring zero phase distortion and in effect doubling the order of the filter. A 1st order high-pass Butterworth filter was then applied with a cutoff frequency of 1 Hz, also using the *filtfilt* function. Bad channels were determined as those whose variance was either less than half or greater than twice that of the surrounding two to four channels for the AM BBN dataset, and three to seven channels for the speech dataset (depending on location). These were then replaced through spherical spline interpolation using EEGLAB (Delorme and Makeig, 2004). Finally, the data were rereferenced to the average of the mastoids, separated into trials based on the triggers provided, and z-scored.

### TRF estimation

The models were fit using TRF estimation implemented via the mTRF Toolbox (Crosse et al., 2016). With TRF estimation, the assumption is that the output EEG, y(t) consists of the convolution of a particular input stimulus feature, x(t) with an unknown system response w(τ) (i.e., the TRF), plus noise (Lalor et al., 2009), i.e.,

y(t)=w(τ)∗x(t)+noise

where τ represents the range of time-lags over which the TRF is estimated. Given the known stimulus feature and the measured EEG, the TRF can be derived (in this case) by performing regularized linear (ridge) regression (for details, see Crosse et al., 2016 ). Baseline correction was performed on each subject’s average TRF (by subtracting the mean value between –20 and 0 ms) before being combined to form the grand average.

### Amplitude binned envelope

The choice of stimulus feature can have a significant influence on the resulting model. Such features could include the envelope (Lalor et al., 2009) or spectrogram (Ding and Simon, 2012; Di Liberto et al., 2015), or in the case of speech, phonemes, phonetic features (Di Liberto et al., 2015), or its semantic content (Broderick et al., 2018). The envelope (time × amplitude) however is probably the most commonly used stimulus feature and is the one chosen for use in this study. For both the AM BBN and speech stimuli the envelopes were calculated by taking the absolute value of their Hilbert transforms, and then resampling them to 128 Hz using the *decimate* function in MATLAB.

As mentioned, it has long been known that the magnitude and latency of auditory system responses vary directly and inversely with stimulus amplitude, respectively, i.e., as the stimulus amplitude increases, the response magnitude increases, and the response latency decreases (and vice versa). Univariate TRFs, like those modeled using envelopes, cannot account for all these amplitude-dependent changes. In fact, univariate TRFs can only account for linear changes in magnitude and cannot account for any changes in latency or morphology. However, simply amplitude binning the envelope (time × [amplitude] × amplitude), i.e., dividing the envelope up into multiple sub-envelopes comprising the different amplitude ranges of the full envelope, normalizing the values in each bin to be between 0 and 1, and then using it to fit a multivariate TRF, should allow the TRF to vary across the different amplitude ranges, potentially enabling it to account for more of these amplitude-dependent changes than its univariate counterpart.

**Figure 2.**
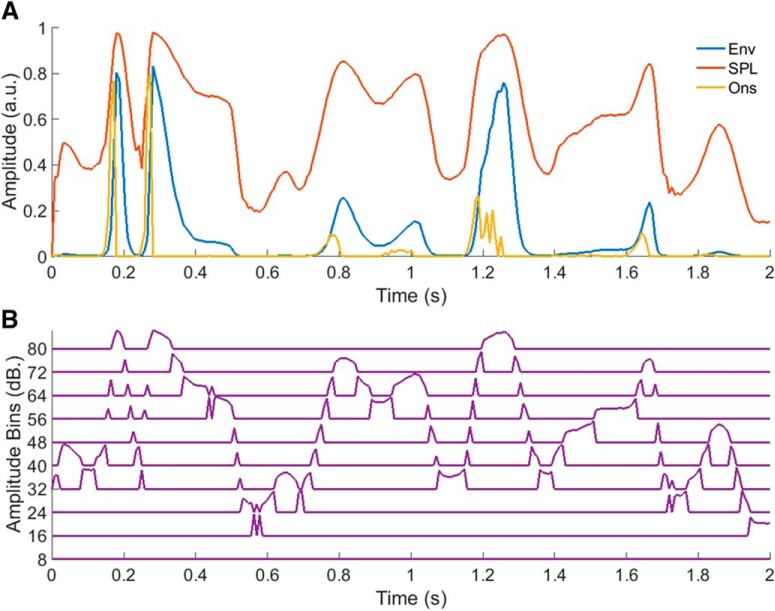
***A***, Example segments of the envelope, SPL envelope, and onset envelope stimulus representations. ***B***, Corresponding segment of the AB envelope.

The amplitude binned (AB) envelope was created by logarithmically binning the envelope into 8 dB bins using the *histcounts* function in MATLAB, and then normalizing the values in each bin to between 0 and 1 (an important step in ensuring the stability of the resulting TRF). This bin size was chosen empirically after comparing the prediction accuracies attained across a range of bin sizes, with broader bins perhaps being less able to capture changes in the response with amplitude, and narrower bins perhaps suffering from the limited amount of data available for training. The logarithmic bin edges were determined by taking 10 to the power of the desired bin edges in dB (i.e., 8, 16, 24, etc.) divided by 20, and then normalizing the resulting range to between 0 and 1 (Fig. 2*B*).

### Other stimulus representations

A number of other approaches have already been put forward that attempt to modify the stimulus representation to account for certain properties of the auditory system. So, rather than just comparing the AB envelope model with the standard envelope model, we also chose to compare it with two others, i.e., the SPL envelope and onset envelope models. The SPL envelope model was fit using an envelope that was transformed into its equivalent logarithmic (SPL) representation, and the onset envelope model was fit using an envelope that was modified to place a greater emphasis on onsets and positive changes in amplitude.

The motivation for using the SPL envelope model derives from the well-known fact that electrophysiological responses generally vary in proportion to the log of the stimulus amplitude (Aiken and Picton, 2008). The SPL envelope was generated by taking 20 times the base 10 logarithm of the envelope (Aiken and Picton, 2008; Fig. 2*A*), and it was hoped that this would help linearize the amplitude to magnitude mapping between the stimulus representation and the EEG. It was presumed that the AB envelope model might outperform the SPL envelope model however, given that they both attempt to account for nonlinearities in the relationship between stimulus amplitude and response magnitude, but only the former accounts for changes in response latency and morphology.

The motivation for using the onset envelope model comes from the idea that many auditory neurons are particularly sensitive to onsets, offsets, and changes in the stimulus (Bieser and Müller-Preuss, 1996), and that this approach has been used effectively in the past (Aiken and Picton, 2008; Hertrich et al., 2012; Fiedler et al., 2017). The onset envelope was explicitly designed to reflect onsets and positive changes in the stimulus, and was created by half-wave rectifying the first-derivative of the envelope (Hertrich et al., 2012; Fig. 2*A*).

### Experimental design and statistical analyses

To compare the different models tested as part of this study, a nested “leave-one-out” cross-validation approach was employed. Specifically, for each stimulus representation, a separate TRF (univariate for the envelope, SPL envelope, and onset envelope, and multivariate for the AB envelope) was fit for each of M trials across several ridge parameters (usually denoted λ) used to regularize the models. One trial was then chosen to be “left out,” i.e., to be used as a “test set,” with the remaining M-1 trials to be used for the inner cross-validation. Of these inner M-1 trials, one trial was again chosen to be left out, i.e., to be used as a “validation set,” with the remaining M-2 trials to be used as a “training set.”

For each λ value, an average model was obtained by averaging over the single-trial models in the training set. These were then convolved with the stimulus representation associated with the validation set to predict its neural response. Model performance was assessed by quantifying how accurately these predicted responses matched the actual recorded response from the validation set, using Pearson’s correlation coefficient. This process was then repeated M-2 times such that each trial was left out of the training set once. The overall model performance was then determined by averaging over the individual model performances for each trial, and the optimal λ value was chosen.

Using this optimal λ value, another average model was then obtained by averaging over the single-trial models in both the training and validation sets. This was then convolved with the stimulus representation associated with the test set to predict its neural response. Model performance was then assessed by quantifying how accurately the predicted response matched the actual recorded response from the test set. This entire procedure was then repeated M-1 times such that each trial was left out of the inner cross-validation procedure once. The overall model performance was then finally determined by averaging over the individual model performances for each trial. Importantly, the parameter optimization was done separately for each stimulus representation and subject, so that we were left comparing each model based on its respective optimal performance.

Again, the performance of each model was assessed by quantifying how accurately the predicted response matched the actual recorded response, using Pearson’s correlation coefficient. The normality of these performance measures for each model was confirmed using the Anderson Darling test, and model comparisons were conducted using paired-sample *t* tests and Cohen’s *d* effect size for paired-sample *t* tests. Cohen’s *d* effect size was calculated by dividing each *t* value by the square-root of the sample size. One potential concern when comparing models with different numbers of parameters is that models with more parameters may perform better simply due to their greater complexity. To account for this, supplementary comparisons were also conducted using the Akaike Information Criterion (AIC) which penalizes models based on their complexity. As the results of these analyses were not normal, model comparisons were conducted using Wilcoxon signed-rank tests.

Permutation tests were also used to assess the null distributions of the envelope models. For the AM BBN dataset, as the stimulus was the same for each trial, a pool of 80 circularly-shifted envelopes (i.e., the original envelope plus 79 circularly-shifted envelopes, each iteratively shifted by 1/80 times the length of the envelope with respect to the previously shifted envelope) were first created. 80 envelopes from this pool were then chosen at random with replacement for use in the cross-validation procedure. This selection and cross-validation procedure was repeated 100 times to determine the null-distribution of the envelope model for each subject. For the speech dataset, as the stimuli were different for each trial, envelopes were simply chosen at random with replacement from the original set of envelopes, for use in the cross-validation procedure. This selection and cross-validation procedure was also repeated 100 times to determine the null-distribution of the envelope model for each subject.

## Results

### Channel selection

EEG prediction accuracies will vary across channels depending on how related the data on those channels are to the stimulus representation. For the AM BBN analyses, the seven channels (of 32) with the highest prediction accuracies for the envelope model were used. For the speech analyses, the 42 channels (of 128) with the highest prediction accuracies for the envelope model, plus three other channels (to ensure symmetry) were used. In both cases, these channels tended to reside over fronto-central to temporal scalp (Di Liberto et al., 2015). The overall prediction accuracy for each model was calculated by averaging the prediction accuracies over these electrodes.

**Figure 3.**
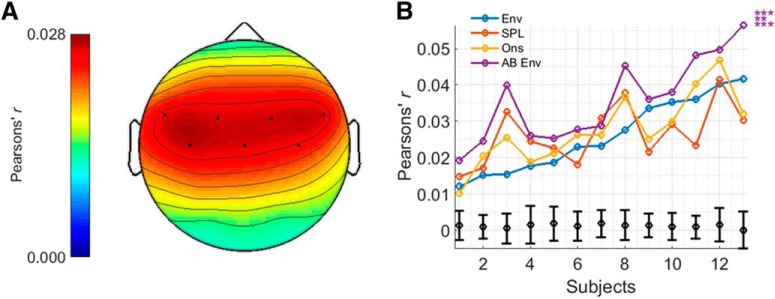
***A***, Topographic plot displaying prediction accuracies for the envelope model for the AM BBN dataset and highlighting the channels chosen for analysis. ***B***, Prediction accuracies for each model and subject, including null hypotheses for the envelope model as determined from the permutation tests (in black), and indications of significance as determined from the *t* tests. Top, middle and bottom rows of asterisks indicate comparisons between AB Env and Env, SPL, and Ons, respectively. ***p < 0.001, **p < 0.01, *p < 0.05.

### Individual model comparisons

#### AM BBN

For the AM BBN dataset, prediction accuracies were determined for each model, and each subject (Fig. 3). All four stimulus representations (i.e., envelope, SPL envelope, onset envelope, and AB envelope) and their associated models were able to predict EEG responses with an accuracy that was significantly above 0.0012, i.e., the null hypothesis obtained using the permutation tests, for all subjects (*t*_(12)_, all *p* < 0.001), and greater than all values obtained using the permutation tests. However, the AB envelope model significantly outperformed all three of the other models, in each case with a large to very large positive effect size (*t*_(12)_ = 5.471, *p* < 0.001, *d* = 1.518 vs the envelope model; *t*_(12)_ = 4.070, *p* < 0.01, *d* = 1.129 vs the SPL envelope model; *t*_(12)_ = 4.800, *p* < 0.001, *d* = 1.331 vs the onset envelope model). These results were also seen when comparing the models using AIC (*p* < 0.001; Wilcoxon signed-rank test). Neither the SPL envelope nor onset envelope models managed to outperform the standard envelope model (*t*_(12)_, both *p* > 0.05).

**Figure 4.**
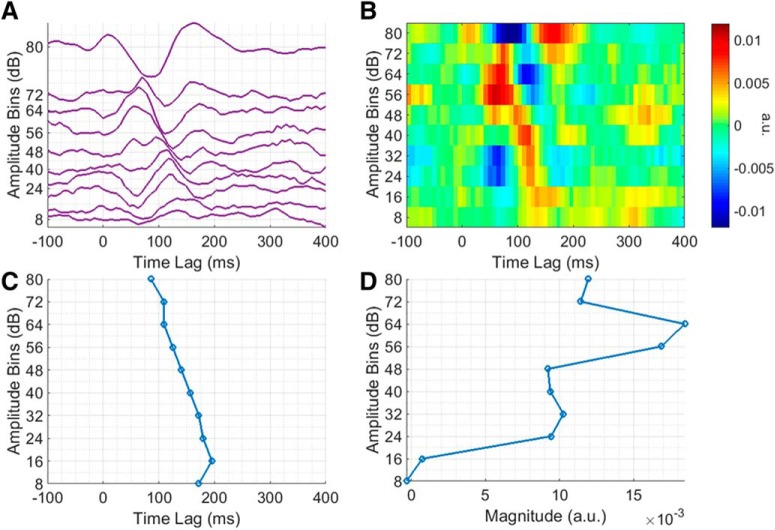
Analysis of amplitude-dependent changes at a single representative channel over left central scalp for the AM BBN dataset. ***A***, Group average AB envelope TRF, plotted to minimize the difference between adjacent traces. ***B***, Image plot of group average AB envelope TRF. ***C***, N1 peak latencies across group average AB envelope TRF bins. ***D***, P1-N1 peak-to-peak amplitudes across group average AB envelope TRF bins.

Exactly how the TRF changes as a function of stimulus amplitude becomes more apparent on closer inspection of the AB envelope TRF (Fig. 4*A*). As the stimulus amplitude decreases, the TRF magnitude decreases, latency increases, and morphology changes in accordance with our hypothesis. The influence of stimulus amplitude on TRF latency is perhaps better emphasized in Figure 4*B*. For example, the “N1”, which is quite large in magnitude in the uppermost amplitude bin, decreases in magnitude and increases in latency, with decreasing stimulus amplitude. To quantify this relationship, the N1 peak in each bin was determined as being the largest negative peak in the TRF at lags between 70 and 210 ms (the corresponding latencies can be seen in Fig. 4*C*). A line was then fit to the data (*R* ^2^ = 0.9143, *p* < 0.001), which showed that the N1 peak latency increases by ∼11 ms with every unit decrease in amplitude bin. A similar effort was made to quantify the relationship between stimulus amplitude and TRF magnitude, i.e., the “P1” peak in each bin was determined as being the largest positive peak in the TRF at lags between 0 and 130 ms (the corresponding P1-N1 peak-peak amplitudes can be seen in Fig. 4*D*). However, while there does seem to be some relationship between stimulus amplitude and TRF magnitude, it was not well fit by a line (*R* ^2^ = 0.597, *p* < 0.01).

#### Speech

**Figure 5.**
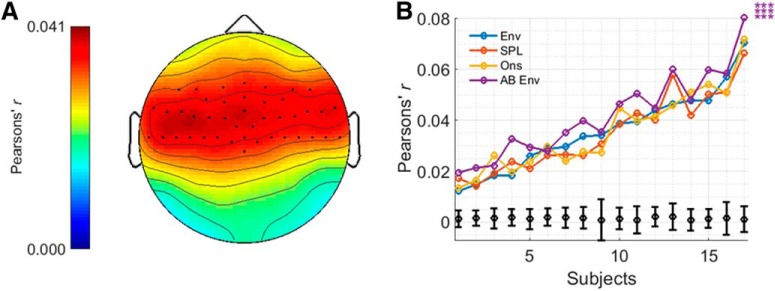
***A***, Topographic plot displaying prediction accuracies for the envelope model for the speech dataset and highlighting the channels chosen for analysis. ***B***, Prediction accuracies for each model and subject, including null hypotheses for the envelope model as determined from the permutation tests (in black), and indications of significance as determined from the *t* tests. Top, middle and bottom rows of asterisks indicate comparisons between AB Env and Env, SPL, and Ons, respectively. ***p < 0.001, **p < 0.01, *p < 0.05.

For the speech dataset, prediction accuracies were again determined for each model, and each subject, with very similar results to before (Fig. 5). All four stimulus representations and their associated models were able to predict EEG responses with an accuracy that was significantly above 0.0015, i.e., the null-hypothesis obtained using the permutation tests, for all subjects (*t*_(16)_, all *p* < 0.001), and greater than all values obtained using the permutation tests. The AB envelope model significantly outperformed all three of the other models, in each case with a large to very large positive effect size (*t*_(16)_ = 5.472, *p* < 0.001, *d* = 1.327 vs the envelope model; *t*_(16)_ = 7.649, *p* < 0.001, *d* = 1.855 vs the SPL envelope model; *t*_(16)_ = 4.666, *p* < 0.001, *d* = 1.132 vs the onset envelope model). These results were also seen when comparing the models using AIC (*p* < 0.001; Wilcoxon signed-rank test). Neither the SPL envelope nor onset envelope models managed to outperform the standard envelope model (*t*_(16)_, both *p* > 0.05).

**Figure 6.**
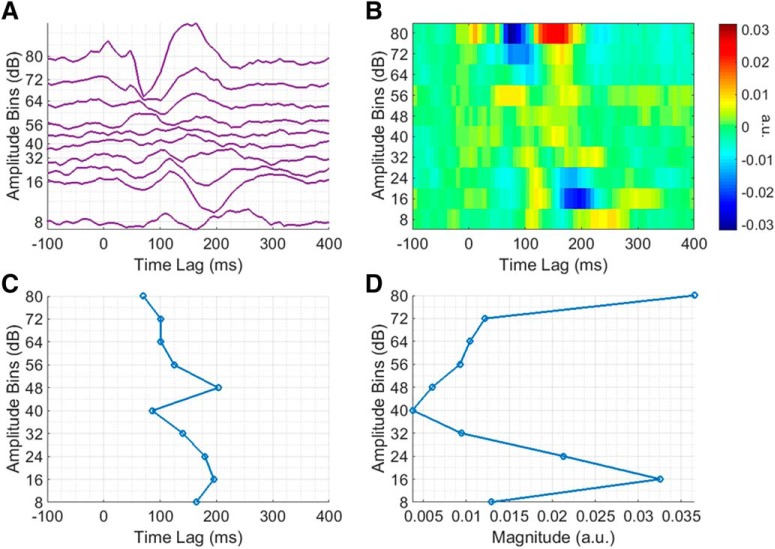
Analysis of amplitude-dependent changes at a single representative channels over left central scalp for the speech dataset. ***A***, Group average AB envelope TRF, plotted to minimize the difference between adjacent traces. ***B***, Image plot of group average AB envelope TRF. ***C***, N1 peak latencies across group average AB envelope TRF bins. ***D***, P1-N1 peak-to-peak amplitudes across group average AB envelope TRF bins.

Again, exactly how the TRF changes as a function of stimulus amplitude becomes more apparent on closer inspection of the AB envelope TRF (Fig. 6*A*,*B*). While the overall relationship between stimulus amplitude and TRF magnitude, latency, and morphology appears similar to before, in this case, the magnitude of the TRF for some of the lower amplitude bins seems unexpectedly high. It is not entirely clear why this would have been the case. The P1 and N1 peaks were also determined in the same manner as before (and the corresponding N1 latencies and “P1-N1” peak-peak amplitudes can be seen in Fig. 6*C*,*D*, respectively). To quantify the relationship between stimulus amplitude and TRF latency, a line was fit to the N1 latency data (*R* ^2^ = 0.5008, *p* < 0.05), which again showed that the N1 peak latency increases by ∼11 ms with every unit decrease in amplitude bin. However, there was no simple relationship between stimulus amplitude and TRF magnitude.

### Combined model comparisons

The comparison between the AB envelope and onset envelope models is not necessarily as straightforward as one might expect. This is because each model is likely reflecting different envelope tracking mechanisms in the cortex (Bieser and Müller-Preuss, 1996). Specifically, the onset envelope model likely reflects contributions from neurons that track onsets and positive changes in amplitude while the AB envelope model likely reflects contributions from neurons that track along with all of the amplitude fluctuations (Bieser and Müller-Preuss, 1996).

**Figure 7.**
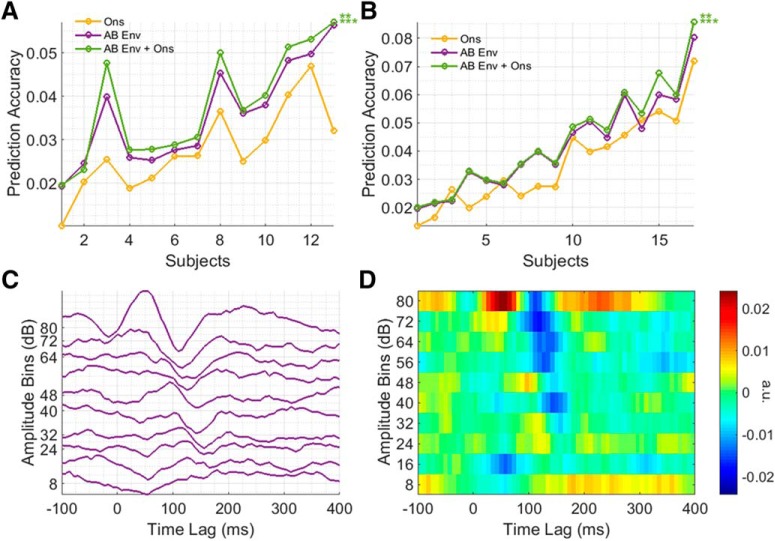
***A***, Prediction accuracies for each model and subject for the AM BBN dataset. ***B***, Prediction accuracies for each model and subject for the speech dataset. Top and bottom rows of asterisks indicate comparisons between AB Env + Ons and AB Env, and Ons, respectively. ***p < 0.001, **p < 0.01. ***C***, Group average AB onset envelope TRF for the AM BBN dataset, plotted to minimize the difference between adjacent traces. ***D***, Image plot of group average AB onset envelope TRF for the AM BBN dataset.

To test the idea that these two models are capturing complementary information on envelope tracking, we investigated whether there would be any advantage in combining these two models (i.e., by combining the two stimulus representations and then using that to fit a multivariate TRF). Indeed, the combined AB envelope plus onset envelope model significantly outperformed the individual onset envelope and AB envelope models, for both the AM BBN (*t*_(12)_ = 5.717, *p* < 0.001, *d* = 1.586 vs the onset envelope; *t*_(12)_ = 4.184, *p* < 0.01, *d* = 1.161 vs the AB envelope) and speech datasets (*t*_(16)_ = 6.139, *p* < 0.001, *d* = 1.489 vs the onset envelope; *t*_(16)_ = 3.312, *p* < 0.01, *d* = 0.8032 vs the AB envelope), suggesting that they are capturing complementary information on envelope tracking in the cortex (Fig. 7*A*,*B*). These results were also seen when comparing the models using AIC (all *p* < 0.001; Wilcoxon signed-rank test).

One obvious extension to this approach then might be to also amplitude bin the onset envelope representation, producing an “AB onset envelope” model. However, while this AB onset envelope TRF exhibits a similar dependence on stimulus amplitude to the AB envelope TRF (Fig. 7*C*,*D*) and significantly outperformed the onset envelope model alone for the AM BBN dataset (*t*_(12)_ = 3.887, *p* < 0.05, *d* = 1.078) although not for the speech dataset (*t*_(16)_, *p* > 0.05), the combined AB envelope plus AB onset envelope model failed to outperform the combined AB envelope plus onset envelope model for either the AM BBN or speech datasets.

## Discussion

Despite it long being known that the latency and morphology (and not just the magnitude) of auditory system responses are dependent on the stimulus amplitude, this has been overlooked in previous efforts at linearly modeling the auditory system. Here we have shown that by allowing the stimulus-response model to vary as a function of the stimulus amplitude, we can improve the modeling of responses to continuous auditory stimuli.

Specifically, we saw that by amplitude binning the envelope and then using that to fit a multivariate TRF, we could improve the prediction accuracy over the standard envelope model with a very large effect size for both the AM BBN and speech datasets. This was not the case for the SPL envelope or onset envelope models however, which both failed to outperform the standard envelope model. We also evaluated the offset envelope (created by half-wave rectifying the negative portion of the first-derivative of the envelope and then using that to fit a univariate TRF) and derivative envelope models but again, neither managed to outperform the envelope model for either dataset and indeed mostly performed worse. Finally, we saw that by combining the AB envelope and onset envelope models, we could further improve the prediction accuracy over the AB envelope model with a large effect size for both the AM BBN and speech datasets.

Interestingly, despite having lower prediction accuracies overall, the improvement in prediction accuracy was greater for the AM BBN dataset. This is likely due to the differences in amplitude distribution seen between the two types of stimuli (Fig. 1*E*,*F*). While the speech stimuli predominantly vary within a narrow amplitude range, the wider “active” amplitude range of the AM BBN stimulus may allow it to benefit more from taking amplitude-dependent variations into account. The reason that the prediction accuracies were higher for the speech dataset overall is likely due to attention effects, e.g., as seen in O’Sullivan et al. (2015), albeit it in that case with two competing speech streams and reconstruction accuracy.

Previous work has shown that the use of other stimulus representations can also improve modeling performance. For example, for speech it has been shown that models based on spectrograms, phonemes, and phonetic features, outperform those based on the standard envelope (Di Liberto et al., 2015). However, for each of these stimulus representations, the same assumption of unchanging TRF morphology applies. For categorical representations such as those reflecting the phonemic/phonetic content of the speech, this could be considered a strength, but for lower-level representations such as the spectrogram, this could be considered a weakness. In the same way as we have done for the envelope in this study, an amplitude binning approach could also be applied to the spectrogram representation of speech by binning the stimulus in each frequency band (time × [frequency × [amplitude]] × amplitude), and then using it to fit a multivariate TRF. This could then potentially account for both amplitude-dependent and frequency-dependent changes in the response, which could lead to improved model performance. Furthermore, as before, the onset envelope could also be included to explain even more of the variance. That said, it should be noted that this representation would be high dimensional and so would come with increased computational requirements as well as an increased chance of overfitting.

Researchers interested in improving the performance of their envelope tracking measures could benefit from using the AB envelope approach and/or including the onset envelope as part of their stimulus representation. The sensitivity and robustness of such measures could be further improved, however, if this work was adapted into a “decoding” framework. Such approaches have become quite popular in recent years and often involve mapping backwards from the multivariate neural data to reconstruct an estimate of the univariate speech envelope that caused those data (O’Sullivan et al., 2015). This approach takes advantage of the large increase in modeling performance that comes with incorporating all of the neural data simultaneously in one multivariate mapping. This stands in contrast with the forward channel-by-channel modeling approach we have used in the present study. As such, it would be practically valuable to incorporate the AB envelope approach into a multivariate-to-multivariate decoding framework. While we have not done that here, such frameworks have been implemented before for multivariate auditory stimuli (Mesgarani et al., 2009), and there are several flexible methods available that would be well suited to such a task (de Cheveigné et al., 2018).

Finally, we suggest that the results of our study should factor into theories on the generative mechanisms underlying the cortical tracking of acoustic envelopes. There are at least two such theories. One proposes that intrinsic, ongoing oscillatory brain rhythms “entrain” to the rhythms of the speech signal by aligning their phase with the stimulus in an anticipatory, behaviorally effective manner (Giraud and Poeppel, 2012; Rimmele et al., 2018). An alternative idea is that cortical tracking of speech (or any auditory stimulus for that matter) occurs as a result of the stimulus providing a driving input to auditory cortex that evokes transient responses in neuronal populations that are tuned to the features of that stimulus and that scale with the strength of those features (Parker and Newsome, 1998). It is well known that sensory neurons are tuned to certain features of the stimuli that they encounter – including features such as frequency and intensity in the auditory domain (Phillips and Irvine, 1981). As such, researchers have explicitly modeled cortical tracking of the speech envelope as a series of transient responses to changes in that speech envelope (Aiken and Picton, 2008). Indeed, this assumption is at the core of the TRF analysis used in this paper. Moreover, in other work, we have shown that EEG responses to continuous speech are well modeled as a series of transient responses to changes in frequency and phonetic features within the speech (Di Liberto et al., 2015). While the present study cannot definitively adjudicate between oscillatory entrainment and transient evoked responses as the underlying mechanism, and indeed maybe both are at play, we do suggest that the relationship we have shown between stimulus amplitude and response latency needs to be considered when positing one or other of these mechanisms. Do smaller amplitude changes evoke later and slower transient responses? Or do they entrain slower oscillations? Or some combination of the two? Work in our lab has begun to look directly at this issue (Lalor, 2019) and will continue to do so.

In summary, here, we have shown that by allowing the stimulus-response model to vary as a function of the stimulus amplitude, we can improve the modeling of responses to continuous auditory stimuli, and that the inclusion of an onset stimulus representation can improve this performance even further. This obviously has implications for how people model auditory processing in humans, but, more generally, points to the importance of incorporating stimulus dependencies when modeling the activity of sensory systems.

## Synthesis

Reviewing Editor: Tatyana Sharpee, The Salk Institute for Biological Studies Decisions are customarily a result of the Reviewing Editor and the peer reviewers coming together and discussing their recommendations until a consensus is reached. When revisions are invited, a fact-based synthesis statement explaining their decision and outlining what is needed to prepare a revision will be listed below. The following reviewer(s) agreed to reveal their identity: Jonas Obleser. Note: If this manuscript was transferred from JNeurosci and a decision was made to accept the manuscript without peer review, a brief statement to this effect will instead be what is listed below.

The authors test whether neural responses to sound amplitude envelopes, as measured in EEG, are better predicted by models that assume a fully linear relationship between stimulus and response magnitudes, or a model that also allows for variation in response morphology (latency, form) at different stimulus amplitudes. They compare several models for non-speech noise stimuli and speech and propose that their findings have implications for encoding of other types of stimulus features as well as for theories of cortical amplitude envelope tracking. As temporal receptive field models are becoming more and more popular, the methodological question asked by this manuscript is timely and important.

The revised manuscript addressed many of the concerns but a number of concerns about statistical analyses remain to be addressed. In particular, degrees of freedom still need to be added in a number of places and most importantly *separate test datasets* need to be used for model evaluation.

1) The data sets for finding model parameters and evaluating model results cannot be the same

2) p. 13, line 292: “... significantly above zero for all subjects (all p < 0.001; t-test), and greater than all values obtained using the permutation tests” Please compare to the value that represents the null-hypothesis and report degrees of freedom.

3) Please report the significance of the permutation tests as e.g. 'and significantly above chance (permutation - p < x)

4) p. 14, line 309: please provide statistical details.

5) Line 230: The statement “(Incidentally, we also experimented with modeling based on envelope offsets and changes without success, please see discussion).” is not suitable for a results section. Either more details on this “experiments” (somewhat sloppy terminology) should be added or reference to these should be removed.

6) Line 429: “An alternative idea is that cortical tracking of speech (or any auditory stimulus for that matter) occurs as a result of the stimulus providing a driving input...” No citations are given for this important idea. Please add key references.

7) Figure 3 contains bad cropping (e.g. of scalp topography manequin).

## References

[B1] Aertsen A, Johannesma PI (1981) The spectro-temporal receptive field. A functional characteristic of auditory neurons. Biol Cybern 42:133–143. 10.1007/BF00336731 7326288

[B2] Aiken SJ, Picton TW (2008) Human cortical responses to the speech envelope. Ear Hear 29:139–157. 18595182 10.1097/aud.0b013e31816453dc

[B3] Beagley HA, Knight JJ (1967) Changes in auditory evoked response with intensity. J Laryngol Otol 81:861–873. 6036752 10.1017/s0022215100067815

[B4] Bieser A, Müller-Preuss P (1996) Auditory responsive cortex in the squirrel monkey: neural responses to amplitude-modulated sounds. Exp Brain Res 108:273–284. 8815035 10.1007/BF00228100

[B5] Boynton GM, Engel SA, Glover GH, Heeger DJ (1996) Linear systems analysis of functional magnetic resonance imaging in human V1. J Neurosci 16:4207–4221. 8753882 10.1523/JNEUROSCI.16-13-04207.1996 PMC6579007

[B6] Broderick MP, Anderson AJ, Liberto GMD, Crosse MJ, Lalor EC (2018) Electrophysiological correlates of semantic dissimilarity reflect the comprehension of natural, narrative speech. Curr Biol 28:803–809.e3. 10.1016/j.cub.2018.01.080 29478856

[B7] Carandini M, Demb JB, Mante V, Tolhurst DJ, Dan Y, Olshausen BA, Gallant JL, Rust NC (2005) Do we know what the early visual system does? J Neurosci 25:10577–10597. 10.1523/JNEUROSCI.3726-05.2005 16291931 PMC6725861

[B8] Chichilnisky EJ (2001) A simple white noise analysis of neuronal light responses. Netw Comput Neural Syst 12:199–213. 10.1080/net.12.2.199.213 11405422

[B9] Crosse MJ, Di Liberto GM, Bednar A, Lalor EC (2016) The multivariate temporal response function (mTRF) toolbox: a MATLAB toolbox for relating neural signals to continuous stimuli. Front Hum Neurosci 10:604. 27965557 10.3389/fnhum.2016.00604 PMC5127806

[B10] David SV, Mesgarani N, Shamma SA (2007) Estimating sparse spectro-temporal receptive fields with natural stimuli. Netw Comput Neural Syst 18:191–212. 10.1080/09548980701609235 17852750

[B11] de Cheveigné A, Wong DDE, Di Liberto GM, Hjortkjær J, Slaney M, Lalor E (2018) Decoding the auditory brain with canonical component analysis. NeuroImage 172:206–216. 10.1016/j.neuroimage.2018.01.033 29378317

[B12] Delorme A, Makeig S (2004) EEGLAB: an open source toolbox for analysis of single-trial EEG dynamics including independent component analysis. J Neurosci Methods 134:9–21. 10.1016/j.jneumeth.2003.10.009 15102499

[B13] Depireux DA, Simon JZ, Klein DJ, Shamma SA (2001) Spectro-temporal response field characterization with dynamic ripples in ferret primary auditory cortex. J Neurophysiol 85:1220–1234. 10.1152/jn.2001.85.3.1220 11247991

[B14] Di Liberto GM, O’Sullivan JA, Lalor EC (2015) Low-frequency cortical entrainment to speech reflects phoneme-level processing. Curr Biol 25:2457–2465. 10.1016/j.cub.2015.08.030 26412129

[B15] Ding N, Simon JZ (2012) Neural coding of continuous speech in auditory cortex during monaural and dichotic listening. J Neurophysiol 107:78–89. 10.1152/jn.00297.2011 21975452 PMC3570829

[B16] Fiedler L, Wöstmann M, Graversen C, Brandmeyer A, Lunner T, Obleser J (2017) Single-channel in-ear-EEG detects the focus of auditory attention to concurrent tone streams and mixed speech. J Neural Eng 14:036020 10.1088/1741-2552/aa66dd 28384124

[B17] Giraud AL, Poeppel D (2012) Cortical oscillations and speech processing: emerging computational principles and operations. Nat Neurosci 15:511. 10.1038/nn.3063 22426255 PMC4461038

[B18] Gonçalves NR, Whelan R, Foxe JJ, Lalor EC (2014) Towards obtaining spatiotemporally precise responses to continuous sensory stimuli in humans: a general linear modeling approach to EEG. Neuroimage 97:196–205. 10.1016/j.neuroimage.2014.04.012 24736185

[B19] Hertrich I, Dietrich S, Trouvain J, Moos A, Ackermann H (2012) Magnetic brain activity phase-locked to the envelope, the syllable onsets, and the fundamental frequency of a perceived speech signal. Psychophysiology 49:322–334. 10.1111/j.1469-8986.2011.01314.x 22175821

[B20] Hubel DH, Wiesel TN (1962) Receptive fields, binocular interaction and functional architecture in the cat’s visual cortex. J Physiol 160:106–154. 10.1113/jphysiol.1962.sp006837 14449617 PMC1359523

[B21] Lalor EC (2019) Evoked activity plays a very substantial role in the cortical tracking of natural speech. Annual Meeting of the Cognitive Neuroscience Society, March 23–26, San Francisco, CA. Abstract C111.

[B22] Lalor EC, Foxe JJ (2010) Neural responses to uninterrupted natural speech can be extracted with precise temporal resolution. Eur J Neurosci 31:189–193. 10.1111/j.1460-9568.2009.07055.x 20092565

[B23] Lalor EC, Power AJ, Reilly RB, Foxe JJ (2009) Resolving precise temporal processing properties of the auditory system using continuous stimuli. J Neurophysiol 102:349–359. 10.1152/jn.90896.2008 19439675

[B24] Liégeois-Chauvel C, Lorenzi C, Trébuchon A, Régis J, Chauvel P (2004) Temporal envelope processing in the human left and right auditory cortices. Cereb Cortex 14:731–740. 10.1093/cercor/bhh033 15054052

[B25] Machens CK, Wehr MS, Zador AM (2004) Linearity of cortical receptive fields measured with natural sounds. J Neurosci 24:1089–1100. 10.1523/JNEUROSCI.4445-03.2004 14762127 PMC6793584

[B26] Mesgarani N, David SV, Fritz JB, Shamma SA (2008) Phoneme representation and classification in primary auditory cortex. J Acoust Soc Am 123:899–909. 10.1121/1.2816572 18247893

[B27] Mesgarani N, David SV, Fritz JB, Shamma SA (2009) Influence of context and behavior on stimulus reconstruction from neural activity in primary auditory cortex. J Neurophysiol 102:3329–3339. 10.1152/jn.91128.2008 19759321 PMC2804432

[B28] O’Sullivan JA, Power AJ, Mesgarani N, Rajaram S, Foxe JJ, Shinn-Cunningham BG, Slaney M, Shamma SA, Lalor EC (2015) Attentional selection in a cocktail party environment can be decoded from single-trial EEG. Cereb Cortex 25:1697–1706. 24429136 10.1093/cercor/bht355 PMC4481604

[B29] Parker AJ, Newsome WT (1998) Sense and the single neuron: probing the physiology of perception. Annu Rev Neurosci 21:227–277. 10.1146/annurev.neuro.21.1.227 9530497

[B30] Phillips DP, Irvine DR (1981) Responses of single neurons in physiologically defined primary auditory cortex (AI) of the cat: frequency tuning and responses to intensity. J Neurophysiol 45:48–58. 10.1152/jn.1981.45.1.48 7205344

[B31] Rimmele JM, Morillon B, Poeppel D, Arnal LH (2018) Proactive sensing of periodic and aperiodic auditory patterns. Trends Cogn Sci 22:870–882. 10.1016/j.tics.2018.08.003 30266147

[B32] Theunissen FE, David SV, Singh NC, Hsu A, Vinje WE, Gallant JL (2001) Estimating spatio-temporal receptive fields of auditory and visual neurons from their responses to natural stimuli. Netw Comput Neural Syst 12:289–316. 10.1088/0954-898X/12/3/304 11563531

[B33] Wu MK, David SV, Gallant JL (2006) Complete functional characterization of sensory neurons by system identification. Annu Rev Neurosci 29:477–505. 10.1146/annurev.neuro.29.051605.113024 16776594

